# Validation of the Chinese version of the adverse life experiences scale

**DOI:** 10.3389/fped.2024.1403183

**Published:** 2024-07-10

**Authors:** Luowei Zhao, Yuling Li, Zhilin Wang, Jie Wu

**Affiliations:** ^1^ Faculty of Psychology, Tianjin Normal University, Tianjin, China; ^2^ School of Teacher Education, Shandong University of Aeronautics, Shandong, China; ^3^ School of Education, Binzhou Polytechnic, Shandong, China; ^4^ Mental Health Education and Research Center, Nanjing Medical University, Jiangsu, China

**Keywords:** adverse childhood experiences, mental health problem, assessment, developmental timing, intergenerational transmission

## Abstract

**Background:**

Adverse childhood experiences (ACEs) are strongly linked to many mental health problems, and play important role in the intergenerational transmission of psychopathology. Additionally, the developmental timing may also be critical in ACEs' impact on these problems. The Adverse Life Experiences Scale (ALES), as a recently developed measure, has demonstrated good reliability and validity in indexing cumulative risk, developmental timing, and intergenerational transmission. This scale has not been used in China. The purpose of present study was to revise the Chinese version of the ALES and examine its psychometric properties.

**Methods:**

A total of 527 parents (fathers *n* = 246, mothers *n* = 281) from families with at least one child (12–18 years) completed this online survey. Internal consistency, test–retest reliability, correlations, regression models were examined for assessing the psychometric properties of the Chinese version of the ALES.

**Results:**

The Chinese version of the ALES showed acceptable internal consistency (children: *α* = .72, parents: *α* = .74) and test–retest reliability (children: *r *= .86, parents: *r* = .84). In terms of validity, both parents and children's ACEs scores (total score and most age intervals scores) were significantly correlated with their current symptoms; ACEs scores of some age intervals in early childhood and adolescence significantly predicted symptoms in regression models; and parents' ACEs total score significantly correlated with children's ACEs total score and symptoms (all, girls, boys) except boys' Strengths and Difficulties Questionnaire total score.

**Conclusion:**

The Chinese version of the ALES showed good psychometric properties for assessing ACEs cumulative risk, developmental timing, and intergenerational transmission, and can serve as a reliable tool to evaluate ACEs in Chinese samples.

## Introduction

Adverse Childhood Experiences (ACEs) include a range of traumatic events that could directly or potentially impede healthy development during the 0–18 years, which primarily consist of child abuse, neglect, domestic violence, severe disruptions of parenting, and other factors related to increased risk for negative health outcomes. These events are highly interrelated, and when considering the cumulative risk, ACEs prove to be a robust predictor of physical and psychosocial problems (1). Although there are some variations in the relationships between ACEs and health problems, the long-term effects are generally consistent across different cultures (2, 3).

Much evidence supports the strong dose-response relationship between ACEs and mental health problems. As a result, the assessment of ACEs in prevalent studies is commonly based on cumulative scoring of ACEs, the validity of which has been well discussed. Other emerging evidence suggests that there may be synergistic effects between different ACEs, and that some specific combinations of ACEs synergistically increase the risk of negative outcomes significantly beyond what is predicted by cumulative scores. However, the dose-response effect is particularly robust for individuals exposed to multiple ACEs, especially four and more (4, 5). In a word, cumulative scoring of ACEs is a concise and practical measurement.

The developmental timing of ACEs may be critical. The impact of ACEs on children is not equal in different developmental stages, and specific sensitive periods may exist. In addition to considering the exposure timing of ACEs, variations in mental health problems may also depend on the chronicity of ACEs (6). Controlling for the number of cumulative ACEs, the chronicity of ACEs can significantly influence the externalizing symptoms (7). Therefore, ACEs measures require competent temporal sensitivity. Some tools used for assessing childhood maltreatment possess the capability to gather temporal information of exposure (8, 9). However, in addition to maltreatment, ACEs also include other important types of childhood adversity, and current measures of ACEs have limited capacity to provide temporal information of exposure. The effects of ACEs in the intergenerational transmission of psychopathology are supported by previous research (10). In general, the measures used to assess caregivers and children ACEs were implemented separately, with the two tools for caregivers and children often being temporarily integrated. Accordingly, recent reviews emphasized the importance of using a unified tool to assess both caregivers and children ACEs (11, 12).

Considering the developmental timing and intergenerational transmission, Hawes and colleagues developed the Adverse Life Experiences Scale (ALES). They also added more qualified ACEs items into this scale based on Felitti and Finkelhor's work (12–14). By employing this scale, researchers can simultaneously assess the level of exposure to ACEs for caregivers and children under the same standard. In Australia, the ALES demonstrated good reliability and validity in measuring both a community sample of families with children aged 2–12 years and a clinical sample of families with children aged 2–9 years. This scale has not been applied to the Chinese sample. With the original author's permission, we revised the Chinese version of the ALES, and replicated the research process of the original scale (community sample part) to examine its cross-cultural psychometric properties.

## Materials and methods

### Participants

Participants were parents from families with at least one child (12–18 years). All participants are anonymous. A total of 527 parents (fathers *n *= 246, mothers *n *= 281) finally completed the survey. Parents were aged between 35 and 58 years (*M *= 44.43; *SD *= 4.55), 53.3% were mothers; children were aged between 12 and 18 years (*M *= 15.05; *SD *= 1.94), 51.6% were girls. Four weeks later, 60 parents were randomly selected to examine the test-retest reliability. Data was collected between April and May 2023.

### Procedure

Participants were recruited from middle schools in Shandong and Yunnan provinces. This survey was conducted via the Wen Juan Xing online survey platform (https://www.wjx.cn). The platform was set up to guarantee that participants input all items and automatically paid each participant 20 Yuan (about $2.8) after they completed the questionnaire. By telephone, we explained to the participants the purpose of the study, the confidentiality of the data, informed them that participation was voluntary and they could quit at any time. The questionnaire link was sent to the participants with their consent. The Tianjin Normal University Ethics Committee approved the procedures (IRB number: 2023033001).

### Measures

#### The adverse life experiences scale (ALES)

The ALES is a caregiver-reported scale that assesses the ACEs of caregivers and their children. Authorized by the original author, the Chinese version of the scale was translated using a standard back-translation procedure. The translation involved two bilingual psychologists, who first translated it from English to Chinese and then back from Chinese to English. The translated Chinese version was cross-checked with the original version to ensure the equivalence of items' meanings. This scale consists of two parallel components with identical items for caregivers and children, each containing 23 risk factor items (yes/no). Considering the cultural differences, some items were adjusted or eliminated to align with the Chinese native culture. In the Chinese version (Appendix A), the items about “dangerous neighborhoods” (1 item) and “war” (2 items) are removed from the original scale, and the item about “bullied by siblings at home” (1 item) is added. The reason for adding the item “bullied by siblings at home” is that studies conducted on Chinese adolescent samples have shown that the proportion of those who experienced sibling bullying was 12.9%–20.9%, and that sibling bullying was significantly associated with mental health problems (15–17). Total score is calculated by summing the “yes” responses across all items (ACEs exposed during 0–18 years), resulting in scores ranging from 0 to 21. In this scale, the entire period of 0–18 years is divided into distinct age intervals to assess the developmental timing of ACEs, including 0–1 year, 2–3 years, 4–5 years, 6–8 years, 9–12 years, 13–18 years. The chronicity of ACEs score is calculated by summing the number of age intervals in which any ACEs item is affirmed and then dividing it by the individuals' age (12). In this study, only parents as caregivers reported ACEs for themselves and their children, and parents only reported the oldest child under 18 years in the household.

#### The strengths and difficulties questionnaire (SDQ)

The SDQ is widely used in the assessment of children's emotional and behavioral problems. The Chinese version of the parent-reported SDQ selected for this study contains 25 items, and consists of five subscales (hyperactivity/inattention, conduct problems, emotional symptoms, peer problems, and prosocial behavior). The SDQ has demonstrated acceptable reliability and validity in Chinese sample studies (18). In this study, the total difficulties score was used and showed good internal consistency (*α* = .75).

#### The child stress disorder checklist (CSDC)

The CSDC is used to assess acute and recent psychopathy responses following traumatic events, and is applicable to children aged 2–18 years (19). This checklist contains 36 items, including 1 item for traumatic events, 5 items for acute reactions, and 30 items for recent responses. In this study, the subscale of recent responses was employed to assess the children's stress symptoms in the past month by calculating the total score. The Chinese version of the CSDC was parent-reported and showed good reliability in the current sample (*α* = .94).

#### The Kessler psychological distress scale (K10)

The scale is widely used to assess the psychological problems in adulthood. In this study, the scale was used to assess parents' anxiety and stress in the past month. The Chinese version of K10 had acceptable internal consistency in the previous study (*α* = .80) (20), and showed good internal consistency in this study (*α *= .94).

### Analytic strategy

The ACEs scores and symptoms were analyzed descriptively. The median ACEs scores of parents and children corresponding to the 50th, 75th, 90th, and 95th percentiles in the sample were counted. The median ACEs scores of parents were counted for ACEs accumulated before 18 years. Since the age range of children is 12–18 years in this study, the median ACEs scores of children were counted for ACEs accumulated before 12 years. Correlations between children's ACEs scores and current symptoms (CSDC, SDQ) were examined. Correlations between parents' ACEs scores and current symptoms (K10) were examined. The predictive power of developmental timing on current symptoms in children and parents was examined using regression models. In the first block, the chronicity scores (independent variable) and age (covariate) were included to test whether the chronicity predicted the current symptoms; in the second block, the ACEs scores (independent variable) corresponding to each age interval were included, to explain the variance in current symptoms beyond the chronicity.

## Results

### Descriptive statistics

The means and standard deviations of ACEs and symptoms are presented in Table 1. In the children's data, the median ACEs scores at the 50th, 75th, 90th, and 95th percentiles of ACEs scores corresponded to one, two, six, and nine; before 12 years, 38.3% of children were exposed to at least one ACE, 9.7% of children were exposed to at least four ACEs. In the parents' data, the median ACEs scores at the 50th, 75th, 90th, and 95th percentiles of ACEs scores corresponded to two, three, six and six; before 18 years, 54.5% of parents were exposed to at least one ACE, 11.6% of parents were exposed to at least four ACEs.

**Table 1:** Descriptive statistics of ACEs and symptoms.

*N* = 527	M	SD
Children ACEs total	1.14	1.80
Children ACEs chronicity	0.07	0.09
Children ACEs 0–1 years	0.09	0.39
Children ACEs 2–3 years	0.08	0.37
Children ACEs 4–5 years	0.13	0.49
Children ACEs 6–8 years	0.25	0.74
Children ACEs 9–12 years	0.44	1.02
Parents ACEs total	1.14	1.88
Parents ACEs chronicity	0.03	0.04
Parents ACEs 0–1 years	0.06	0.27
Parents ACEs 2–3 years	0.13	0.57
Parents ACEs 4–5 years	0.20	0.53
Parents ACEs 6–8 years	0.42	1.03
Parents ACEs 9–12 years	0.47	1.03
Parents ACEs 13–18 years	0.71	1.46
SDQ total score	15.73	5.07
CSDC total score	6.78	7.96
K10 total score	16.45	6.97

### Reliability

The Chinese version of ALES for both children and parents had shown acceptable internal consistency (children: *α* = .72, parents: *α* = .74). Reliabilities for children and parents were both good in four weeks later retest (children: *r *= .86, parents: *r* = .84).

### Validity

The bivariate correlations between ACEs Scores and Symptoms are shown in Table 2. In children, the ACEs total score were significantly correlated with SDQ total score (*r *= .32, *p* < .01) and significantly correlated with CSDC total score (*r *= .45, *p* < .01). For each age interval, the correlation between ACEs and SDQ total score was not significant for 2–3 years, the correlations were significant for 0–1 year (*r* = .13, *p* < .01) and the remaining age intervals (*r* = .25–.30, *p* < .01); the correlations between ACEs and CSDC total score were significant across all age intervals (*r* = .22–.43, *p* < .01). The children's ACEs chronicity was significantly correlated with SDQ total score (*r *= .29, *p* < .01) and significantly correlated with CSDC total score (*r *= .43, *p* < .01).

**Table 2:** Bivariate correlations between ACEs scores and symptoms.

*N* = 527	1	2	3	4	5	6	7	8	9	10	11	12	13	14	15	16	17	18	19
1. SDQ Total score	1																		
2. CSDC Total score	.71**	1																	
3. K10 Total Score	.47**	.49**	1																
4. Children ACEs Total	.32**	.45**	.24**	1															
5. Children ACEs Chronicity	.29**	.43**	.25**	.83**	1														
6. Children ACEs 0–1 years	.13**	.22**	.09*	.38**	.38**	1													
7. Children ACEs 2–3 years	.08	.24**	.08	.53**	.51**	.50**	1												
8. Children ACEs 4–5 years	.25**	.33**	.18**	.72**	.64**	.24**	.69**	1											
9. Children ACEs 6–8 years	.30**	.32**	.15**	.78**	.65**	.17**	.44**	.79**	1										
10. Children ACEs 9–12 years	.26**	.34**	.20**	.80**	.70**	.18**	.42**	.73**	.75**	1									
11. Children ACEs 13–18 years	.30**	.43**	.25**	.86**	.64**	.16**	.32**	.60**	.61**	.64**	1								
12. Parents ACEs Total	.20**	.31**	.39**	.56**	.57**	.28**	.33**	.47**	.47**	.53**	.45**	1							
13. Parents ACEs Chronicity	.28**	.40**	.33**	.55**	.67**	.23**	.32**	.44**	.46**	.45**	.43**	.71**	1						
14. Parents ACEs 0–1 years	−.01	.13**	.08	.28**	.23**	.24**	.26**	.19**	.18**	.22**	.21**	.26**	.41**	1					
15. Parents ACEs 2–3 years	.16**	.17**	.21**	.31**	.34**	.61**	.22**	.24**	.18**	.16**	.19**	.41**	.47**	.29**	1				
16. Parents ACEs 4–5 years	.22**	.29**	.28**	.44**	.51**	.09*	.18**	.42**	.43**	.46**	.38**	.51**	.79**	.39**	.36**	1			
17. Parents ACEs 6–8 years	.23**	.28**	.25**	.58**	.60**	.34**	.32**	.50**	.50**	.53**	.45**	.69**	.65**	.18**	.55**	.54**	1		
18. Parents ACEs 9–12 years	.27**	.34**	.28**	.60**	.60**	.10*	.33**	.58**	.61**	.65**	.50**	.73**	.69**	.17**	.17**	.59**	.68**	1	
19. Parents ACEs 13–18 years	.19**	.26**	.37**	.44**	.41**	.31**	.34**	.42**	.36**	.39**	.38**	.89**	.51**	.13**	.42**	.26**	.54**	.55**	1

* *p *< .05.

** *p* < .01.

*** *p* < .001.

In parents, the ACEs total score were significantly correlated with K10 total score(*r* = .39, *p* < .01). For each age interval, the correlation between ACEs and K10 total score was not significant for 0–1 year, and the correlations were significant for the remaining age intervals (*r* = .21–.37, *p* < .01). The parents' ACEs chronicity was significantly correlated with K10 total score (*r *= .33, *p* < .01).

Regression models for the developmental timing of children's ACEs and their current symptoms are presented in Table 3. In boys, the ACEs chronicity and age did not yield significant predictions for overall symptoms, the ACEs scores corresponding to the age intervals of 2–3 years (*β *= −.20, *p* < .05), 6–8 years (*β *= .30, *p* < .05), 9–12 years (*β *= −.31, *p* < .05) were found to be significant predictors of overall symptoms. Posttraumatic stress symptoms in boys were significantly predicted by ACEs chronicity (*β *= .44, *p* < .001), and the ACEs scores corresponding to the age intervals of 0–1 year (*β *= −.16, *p* < .05), 9–12 years (*β *= −.27, *p* < .05), and 13–18 years (*β *= .24, *p* < .01) were significant predictors of posttraumatic stress symptoms.

**Table 3:** Developmental timing of children's ACEs as predictors of current symptoms.

Predictor variable	Overall symptoms (SDQ total score)						Posttraumatic stress symptoms (CSDC total score)					
	Boys (*n *= 255)			Girls (*n* = 272)			Boys (*n* = 255)			Girls (*n* = 272)		
	B	*SE*	*β*	B	*SE*	*β*	B	*SE*	*β*	B	*SE*	*β*
Block 1												
Age	−.19	.17	−.07	.16	.15	.06	−.11	.24	−.03	.01	0.23	.00
Chronicity	7.82	6.19	.15	11.36	5.44	.19*	33.82	8.48	.44***	28.16	8.63	.27**
Block 2												
0–1 year	.12	.95	.01	2.89	.96	.20**	−2.60	1.30	−.16*	7.05	1.52	.27***
2–3 years	−2.47	1.19	−.20*	−5.12	1.61	−.34**	1.43	1.64	.08	−6.35	2.55	−.24*
4–5 years	1.60	1.55	.16	2.17	1.26	.20	.52	2.12	.04	3.52	2.00	.19
6–8 years	1.96	.95	.30*	.59	.58	.08	−.53	1.30	−.06	−.12	.92	.01
9–12 years	−1.50	.63	−.31*	.23	.42	.05	−1.84	.86	−.27*	.26	.67	.03
13–18 years	.24	.40	.06	.76	.31	.20*	1.44	.55	.24**	1.60	0.49	.24**

* *p *< .05.

** *p* < .01.

*** *p* < .001.

In girls, the ACEs chronicity significantly predicted overall symptoms (*β *= .19, *p* < .05), and the ACEs scores corresponding to the age intervals of 0–1 year (*β *= .20, *p* < .01), 2–3 years (*β *= −.34, *p* < .01), 13–18 years (*β *= .20, *p* < .05) were significant predictors of overall symptoms. Posttraumatic stress symptoms in girls were significantly predicted by the ACEs chronicity (*β *= .27, *p* < .01), and the ACEs scores corresponding to the age intervals of 0–1 year (*β *= .27, *p* < .001), 2–3 years (*β *= −.24, *p* < .05), 13–18 years (*β *= .24, *p* < .01) significantly predicted posttraumatic stress symptoms. Multicollinearity among all age intervals was controlled for in all regression models.

Two regression models for the developmental timing of parents' ACEs and current symptoms are presented in Table 4. In fathers, the ACEs chronicity and age did not yield significant predictions for psychopathy symptoms, the ACEs scores corresponding to the age interval of 13–18 years (*β* = .37, *p* < .001) significantly predicted current symptoms. In mothers, the ACEs chronicity and age failed to predict their psychopathy symptoms, the ACEs scores corresponding to the age intervals of 4–5 years (*β *= .28, *p* < .01) and 13–18 years (*β *= .33, *p* < .001) were significant predictors of current symptoms. Multicollinearity among all age intervals was controlled for in all regression models.

**Table 4:** Developmental timing of parents’ ACEs as predictors of symptoms.

Predictor variable	Psychopathy symptoms (K10 total score)					
	Fathers (*n* = 246)			Mathers (*n* = 281)		
	B	*SE*	*β*	B	*SE*	*β*
Block 1						
Age	.14	.09	.10	−.11	.09	−.07
Chronicity	22.02	21.14	.13	−11.15	28.66	−.05
Block 2						
0–1 year	−1.39	1.32	−.07	−3.82	2.68	−.11
2–3 years	.63	.91	.07	1.11	2.09	.04
4–5 years	1.89	1.11	.17	4.48	1.67	.28**
6–8 years	−.61	.70	−.11	−.45	.75	−.05
9–12 years	−.30	0.75	−.05	.07	.76	.01
13–18 years	1.36	.28	.37***	2.33	.61	.33***

** *p* < .01.

*** *p* < .001.

Intergenerational transmission of psychopathology was examined. Parents' ACEs total scores were significantly correlated with children's ACEs total score (*r* = .56, *p* < .01, *n *= 527), girls' ACEs total score (*r* = .63, *p* < .0, *n *= 272), and boys' ACEs total score (*r *= .51, *p* < .01, *n *= 255). Using Fisher's z transformation for testing, the correlation between parents' ACEs total score and girls' ACEs total score was significantly higher than that of boys (*z *= 2.04, *p* < .05) (21). Parents' ACEs total score demonstrated significant correlations with children's SDQ total score (*r* = .20, *p* < .01, *n *= 527) and girls' SDQ total score (*r* = .46, *p *< .01, *n *= 272), while no significant association was found with boys’ SDQ total score (*n *= 255). Significant correlations were observed between the parents' ACEs total score and children's CSDC total score (*r *= .31, *p* < .01, *n *= 527), girls' CSDC total score (*r* = .56, *p* < .01, *n *= 272), and boys' CSDC total score (*r *= .14, *p* < .05, *n *= 255). Using Fisher's z transformation for testing, the correlation between parents' ACEs total score and girls' CSDC total score was significantly higher than that of boys (*z *= 5.61, *p* < .001) (21).

## Discussion

The results of this study indicate that the Chinese version of the ALES has acceptable reliability and validity, within a sample of Chinese families raising at least one child (12–18 years). Both parent and children ACEs total score, as measured by this scale, were associated with current symptoms, consistent with findings from previous studies. ACEs chronicity and ACEs scores corresponding to the specific age intervals further explained variance in these outcomes. Several ACEs age intervals in early childhood and adolescence significantly predicted symptoms (refer to Tables 3, 4). In intergenerational transmission of psychopathology, parents' ACEs total score significantly correlated with children's ACEs total score and symptoms (all, girls, boys) except boys' SDQ total score, and the correlations were all significantly higher in girls than in boys.

Based on the regression models of children and parents, the results indicate that the impact of ACEs on subsequent psychopathy symptoms is significant in early childhood and adolescence. In other words, these findings suggest that both early childhood and adolescence are sensitive periods for individual responses to adversity. The effects of exposure to stress and adversity during early childhood on long-term bio-behavioral functioning in life-course health development, which surpass the effects observed in other developmental stages, have been extensively discussed (22, 23). Previous findings support that early childhood exposure to ACEs leads to greater variations in amygdala development and DNA methylation patterns, thereby increasing sensitivity in responding to subsequent stress and adversity (24–26). Some studies suggested that adolescence may provide children another opportunity to shape their stress response system (27–29). Adolescence emerges as a sensitive period for stress response due to the synchronous maturation of the endocrine system, neural system, psychosocial functioning, and increased plasticity across multiple systems (30).

In children models, significant negative associations were found between ACEs in some age intervals (under 12 years) and current symptoms. The results may be explained by the stress acceleration theory and cultural contexts. The Stress Acceleration Hypothesis posits that early-life adversity can result in an accelerated maturation of the emotional circuits in the children's brain, thereby facilitating rapid adaptation to stressful environmental stimuli (31). Individuals exposed to adversity during early childhood may exhibit better cognitive performance in stressful or risky tasks, compared to their peers living in supportive environment (32). Accelerated development can ensure conditional adaptation in resource-disadvantaged and uncertain environments, thereby increasing survivability (33, 34). The potential cost associated with the benefits of accelerated development is the alteration in neurodevelopmental plasticity, consequently increasing the risk of developing psychopathy symptoms in adulthood (31, 35, 36). Overall, emerging studies suggest that children exposed to early adversity may exhibit a holistic pattern of accelerated neurodevelopment (37). Due to the influence of multiple factors, the mechanisms by which accelerated neurodevelopment in response to early adversity yields benefits and costs throughout the lifespan are complex (34, 38). From the cultural context perspective, Chinese traditional culture, rooted in Confucianism, emphasizes a positive appraisal of adversity, and believes that successfully coping with adversity is an essential way to promote resilience and enhance self-worth. Studies among Chinese participants (including Hong Kong) have found that children who have experienced poverty show greater resilience, more positive beliefs and behaviors in the face of adversity (39, 40). Furthermore, in Chinese family, virtues of harmony and endurance are underscored, which would help children cope with family conflict (41, 42). Research suggests that balanced sibling relationships, including both conflict and support, are associated with the better social-emotional skills. Therefore, although sibling bullying is significantly associated with mental health problems overall, moderate exposure to sibling bullying in such a family context may potentially have positive effects on children's development (43).

In intergenerational transmission of psychopathology, the results suggest that girls may be more susceptible than boys, indicating the potential moderating role of gender in this process. In a study of the relationship between mothers' exposure to childhood sexual abuse and their children's externalizing behavioral problems, the relationship was found to be significant for mother-girl dyads but not for mother-boy dyads (44). In another study, contrasting findings were observed, suggesting that boys are more susceptible to the indirect effects generated by mothers' ACEs through perinatal anxiety and depression (45). Additionally, in Chinese preschool children sample, no significant gender differences were found in the influence of mothers' ACEs on children's behavioral problems (46). One possible explanation for the above disagreement is that the transmission pathways of parents' ACEs differ between boys and girls, which is supported by the study of genetic epigenetic biomarkers (47). Another explanation is that the contributions of different ACEs types in the intergenerational transmission may be not equal (48).

This study has several limitations. First, it's recommended that participants from diverse demographic backgrounds, such as socioeconomic statuses, ethnicities, and religious beliefs, be included to ensure a more representative sample, especially clinical-related participants. Second, for some specific ACEs, such as “Adult has repeatedly cursed, insulted, threatened to hurt”, parents may act as the perpetrators and intentionally conceal or underreport their children's ACEs. In future studies, the ACEs scores of children (12–18 years) should consider self-report. Third, to avoid participants impatience caused by excessive response time, the number of all items in this survey was controlled. As a result, the collection of symptoms information was relatively inadequate, especially for parents. Additional symptom measures could be included in the future to provide more evidence to support the Chinese version of the ALES.

## Conclusions

In summary, the findings of this study indicate that the Chinese version of ALES has good psychometric properties and can be considered a reliable and valid tool. This scale provides a comprehensive approach to assessing ACEs by considering cumulative score, developmental timing, and intergenerational transmission. Implementation of this scale would contribute to a deeper understanding of the relationship between ACEs and mental health problems, thereby improving current prevention and intervention strategies.

## Data Availability

The original contributions presented in the study are included in the article/Supplementary Material, further inquiries can be directed to the corresponding authors.

## Appendix A. The Chinese version of adverse life experiences scale

### 1 Parents: self-report

The following part is about challenging things sometimes happen in your lives before 18 years. Please select “No” if you have not experienced, “yes” if you have experienced and continued select the age intervals.

### 2 Children: parent-report

The following part is about challenging things sometimes happen in your child lives. Please select “No” if your child have not experienced, “yes” if your child have experienced and continued select the age intervals. If you have more than one child under the age of 18, please report the oldest.

**Table:**

Items	Yes/No	0–1 years	2–3 years	4–5 years	6–8 years	9–12 years	13–18 years
1. Have you been seriously ill or injured or been in a serious accident?	** **	** **	** **	** **	** **	** **	** **
2. Have you missed out on an important part of your education? (e.g., lengthy time away from school, didn't receive necessary learning support)	** **	** **	** **	** **	** **	** **	** **
3. Have you felt lonely, or been rejected or excluded by peers?	** **	** **	** **	** **	** **	** **	** **
4. Have you been hurt, threatened, picked on, or insulted by peers?	** **	** **	** **	** **	** **	** **	** **
5. Have you been hurt, threatened, or bullied by siblings or cousins.	** **	** **	** **	** **	** **	** **	** **
6. Have you been affected by a natural disaster or social public crises? (e.g., pandemic diseases, flood, bushfire, cyclone, or earthquake)	** **	** **	** **	** **	** **	** **	** **
7. Has there been a time when your family was very poor, or experienced serious financial problems?	** **	** **	** **	** **	** **	** **	** **
8. Did you ever not have enough to eat, have to wear dirty clothes, were not taken to a doctor when needed, or were left alone without someone to look after you?	** **	** **	** **	** **	** **	** **	** **
9. Has an adult repeatedly sworn at, insulted, put down, humiliated, or threatened to hurt you?	** **	** **	** **	** **	** **	** **	** **
10. Have you felt that no one in your family loved you or that no one thought you were important?	** **	** **	** **	** **	** **	** **	** **
11. Have you been separated from or lost someone who you depended on for love or security? (e.g., abandonment, immigration, by accident, severe illness, or death)	** **	** **	** **	** **	** **	** **	** **
12. Have you seen a family member get pushed, slapped, hit, punched, kicked, or threatened by another family or household member?	** **	** **	** **	** **	** **	** **	** **
13. Have you lived with someone who has addictive behaviors? (e.g., alcohol, drug, gambling, etc.)	** **	** **	** **	** **	** **	** **	** **
14. Have you lived with someone who was severe illness (including mental illness), or who attempted suicide?	** **	** **	** **	** **	** **	** **	** **
15. Has a family member been arrested, jailed, or taken away by authorities?	** **	** **	** **	** **	** **	** **	** **
16. Have you been discriminated or felt like an outsider? (e.g., due to your gender, residence or school change)	** **	** **	** **	** **	** **	** **	** **
17. Have you been isolated or removed from a community, cultural group, or land?	** **	** **	** **	** **	** **	** **	** **
18. Have you been pushed, grabbed, slapped, or injured by an adult?	** **	** **	** **	** **	** **	** **	** **
19. Have you been forced into sexual acts, or forced to look at sexual things? (e.g., Publications, videos, etc.)	** **	** **	** **	** **	** **	** **	** **
20. Have you seen another person seriously injured or killed, or have you repeatedly heard about others getting hurt or killed?	** **	** **	** **	** **	** **	** **	** **
21. Have you had a sibling, close extended family member or close friend die?	** **	** **	** **	** **	** **	** **	** **

**Table:**

Items	Yes/No	0–1 years	2–3 years	4–5 years	6–8 years	9–12 years	13–18 years
1. Has your child been seriously ill or injured or been in a serious accident?	** **	** **	** **	** **	** **	** **	** **
2. Has your child missed out on an important part of her/his education? (e.g., lengthy time away from school, didn't receive necessary learning support)	** **	** **	** **	** **	** **	** **	** **
3. Has your child felt lonely, or been rejected or excluded by peers?	** **	** **	** **	** **	** **	** **	** **
4. Has your child been hurt, threatened, picked on, or insulted by peers?	** **	** **	** **	** **	** **	** **	** **
5. Has your child been hurt, threatened, or bullied by siblings or cousins.	** **	** **	** **	** **	** **	** **	** **
6. Has your child been affected by a natural disaster or social public crises? (e.g., pandemic diseases, flood, bushfire, cyclone, or earthquake)	** **	** **	** **	** **	** **	** **	** **
7. Has there been a time when your child's family was very poor, or experienced serious financial problems?	** **	** **	** **	** **	** **	** **	** **
8. Was there ever a time when your child did not have enough to eat, had to wear dirty clothes, was left alone without someone to look after him/her, or was not taken to a doctor when needed?	** **	** **	** **	** **	** **	** **	** **
9. Has an adult repeatedly sworn at, insulted, put down, humiliated, or threatened to hurt your child?	** **	** **	** **	** **	** **	** **	** **
10. Has your child felt that no one in your family loved him/her or that no one thought he/she was important?	** **	** **	** **	** **	** **	** **	** **
11. Has your child been separated from or lost someone who he/she depended on for love or security? (e.g., abandonment, immigration, by accident, severe illness, or death)	** **	** **	** **	** **	** **	** **	** **
12. Has your child seen a family member get pushed, slapped, hit, punched, kicked, or threatened by another family or household member?	** **	** **	** **	** **	** **	** **	** **
13. Has your child lived with someone who has addictive behaviors? (e.g., alcohol, drug, gambling, etc.)	** **	** **	** **	** **	** **	** **	** **
14. Has your child lived with someone who was severe illness (including mental illness), or who attempted suicide?	** **	** **	** **	** **	** **	** **	** **
15. Has your child had a family member been arrested, jailed, or taken away by authorities?	** **	** **	** **	** **	** **	** **	** **
16. Has your child been discriminated or felt like an outsider? (e.g., due to the gender, residence or school change)	** **	** **	** **	** **	** **	** **	** **
17. Has your child been isolated or removed from a community, cultural group, or land?	** **	** **	** **	** **	** **	** **	** **
18. Has your child been pushed, grabbed, slapped, or injured by an adult?	** **	** **	** **	** **	** **	** **	** **
19. Has your child been forced into sexual acts, or forced to look at sexual things? (e.g., Publications, videos, etc.)	** **	** **	** **	** **	** **	** **	** **
20. Has your child seen another person seriously injured or killed, or have you repeatedly heard about others getting hurt or killed?	** **	** **	** **	** **	** **	** **	** **
21. Has your child had a sibling, close extended family member or close friend die?							
